# Stand by Me: Qualitative Insights into the Ease of Use of Adjustable Workstations

**DOI:** 10.3934/publichealth.2016.3.644

**Published:** 2016-08-30

**Authors:** Justine Leavy, Jonine Jancey

**Affiliations:** Collaboration for Evidence, Research and Impact in Public Health, School of Public Health, Faculty of Health Science, Curtin University, Australia

**Keywords:** sedentary behaviour, sitting time, adjustable workstations, Sit-to-Stand, qualitative, employee, employer, Oganisational Health Promotion

## Abstract

**Background:**

Office workers sit for more than 80% of the work day making them an important target for work site health promotion interventions to break up prolonged sitting time. Adjustable workstations are one strategy used to reduce prolonged sitting time. This study provides both an employees' and employers' perspective into the advantages, disadvantages, practicality and convenience of adjustable workstations and how movement in the office can be further supported by organisations.

**Methods:**

This qualitative study was part of the Uprising pilot study. Employees were from the intervention arm of a two group (intervention n = 18 and control n = 18) study. Employers were the immediate line-manager of the employee. Data were collected via employee focus groups (n = 17) and employer individual interviews (n = 12). The majority of participants were female (n = 18), had healthy weight, and had a post-graduate qualification. All focus group discussions and interviews were recorded, transcribed verbatim and the data coded according to the content. Qualitative content analysis was conducted.

**Results:**

Employee data identified four concepts: enhanced general wellbeing; workability and practicality; disadvantages of the retro-fit; and triggers to stand. Most employees (n = 12) reported enhanced general well-being, workability and practicality included less email exchange and positive interaction (n = 5), while the instability of the keyboard a commonly cited disadvantage. Triggers to stand included time and task based prompts. Employer data concepts included: general health and wellbeing; work engagement; flexibility; employee morale; and injury prevention. Over half of the employers (n = 7) emphasised back care and occupational health considerations as important, as well as increased level of staff engagement and strategies to break up prolonged periods of sitting.

**Discussion:**

The focus groups highlight the perceived general health benefits from this short intervention, including opportunity to sit less and interact in the workplace, creating an ‘energised’ work environment. The retro-fit workstation and keyboard platform provided challenges for some participants. Supervisors emphasised injury prevention and employee morale as two important by products of the adjustable workstation. These were not mentioned by employees. They called for champions to advocate for strategies to break up prolonged sitting.

**Implications:**

The findings of this novel research from both the employee and employer perspective may support installation of adjustable workstations as one component of a comprehensive approach to improve the long term health of employees.

## 1. Introduction

Office based workers have become increasingly sedentary. This poses a challenge for organisations and public health alike due to the evidence linking sitting time with adverse health outcomes [1]–[4]. Many full-time adult office workers in Australia, the United Kingdom (UK) and the United States (US), sit for more than 80% of their total time at work [1],[4]–[7]. This is problematic as several recent studies have demonstrated that prolonged sedentary behaviour (including sitting) is an independent risk factor for many negative health outcomes [8]–[13]. The importance of breaking up prolonged periods of sitting in office based workers has become increasingly recognised as a priority workplace health issue.

The workplace has been recognised as an important setting to promote physical activity among adults [14],[15] and more recently a key setting to introduce strategies to reduce sitting time to improve health [1],[7],[11],[16]–[18]. Accordingly, recent evidence suggests that by introducing sit-to-stand workstations, periods of extended sitting can be reduced [1],[7],[19]–[23]. These workstations have been reported to have a reasonably high level of acceptability [16],[19], and have been associated with improvement in employee's mood and muscular skeletal disorders [22],[23]. However, the need for modifications to the physical office space when installing sit-to-stand work stations, which may disrupt employees' work routines, can adversely impact on their uptake [4],[7]. This has prompted a call for ‘real world’ research that focuses on implementation issues related to optimising both employers' and employees' uptake of this equipment [4],[20],[24].

Given that the main type of employment in most developed countries is office-based and working adults typically spend a large proportion of their day sitting, this is an appropriate site for interventions aimed at decreasing sitting time [23],[25]. Although published research aimed at replacing sitting time with standing in natural work environments using sit-to-stand workstations has increased over the past five years, including several systematic reviews [26]–[28], only six studies have used in-depth qualitative research [12],[17],[19],[29],[30],[31]. To date the qualitative studies conducted in Australia [12],[19],[29],[30] and Europe [17],[31] have focussed mostly on the employee perspective [17],[19],[29]–[31], with one study examining the perspective of occupational health and safety practitioners [12]. The research concluded that sit-to-stand desks have high acceptability, usability and feasibility [19],[29],[31]. Furthermore, it was recommended that future studies explore: normalising standing for work related tasks [19]; facilitators, practical insights and strategies to target ‘sitting less, moving more’ [17],[30],[31]; and different populations and settings [29]. To date no studies have explored *both* the supervisors' and their employees' views to inform a better understanding of, and insight related to the use of sit-to-stand workstations in an office based setting.

To this end, this qualitative study explores the impact of sit-to-stand workstations that were installed in an office based workplace from the perspective of both the employee (user of the sit-to-stand workstation) and the employee's supervisor (immediate line manager of the employee using the sit-to-stand workstation). This study will provide insights into: the advantages and disadvantages; practicality; ease of use; and convenience of the adjustable workstations from both an employee, and an employer perspective (referred to as supervisors in this study) in office based workers located in Perth, Western Australia; and how movement in the office can be further supported.

## 2. Methods

The qualitative evaluation is one component of the Uprising pilot study, to explore sitting and standing time in the workplace after the installation of adjustable workstations. Full details of the study are provided elsewhere [18]. Focus group discussions (FGD) and individual interviews (II) were conducted [32]. Ethics approval for the Uprising Study was granted by Curtin University's Human Research Ethics Committee (approval number SPH-37-13).

### 2.1. Study Design and Participants

The employee sample was drawn from the intervention arm of a two group (intervention n = 18 and control n = 18) study. The Ergotron model Workfit-A single screen on an articulated arm or Workfit-S dual screen on a pedestal stand (www.ergotorn.com) [33] were installed for a four week intervention period. Employees were provided with instructions by a physiotherapist on the first day after the installation of the workstation and a brief educational intervention [34] was delivered by a research team member (JL) to all intervention participants. Permission to install the workstations was obtained from the employee's supervisor (immediate line manager). The employee inclusion criteria required participants to have a desk based job, no recent physical injuries or discomfort, aged between 25 to 54 years, and work at least four days a week.

The supervisor was responsible for approving their employee's participation in the intervention and permission for the employee's work desk to be modified to accommodate the adjustable workstation. After the study period supervisors were sent an email inviting them to undertake an individual interview with a trained researcher at a time convenient to them.

### 2.2. Focus Groups and Interviews

A discussion guide was developed for the focus groups with employees and an interview schedule for the individual interviews with the supervisors. Focus groups were chosen as a mechanism for helping employees generate and share their insights, and time and budget constraints prohibited individual employee interviews. Individual interviews were chosen for the supervisors as it was preferable to collect responses without the potential for the employee group influence factor, and logistically easier to conduct one-on-one due to work schedules. The two methods were chosen as they are complementary. The focus groups were conducted in meeting rooms on the University campus, during the period of June to October, 2014. The interviews were conducted at the work places of the supervisors or by telephone, during the period of July-November, 2014. Prior to conducting the focus groups and the interviews, the purpose of the study was explained and participants' informed consent for their involvement in the research was obtained.

### 2.3. Employee Focus Groups

Focus groups with the employees were conducted two weeks after the completion of the study (removal of the sit-to-stand workstation), and were facilitated by two members of the research team. One researcher facilitated each focus group discussion (JJ), while the other took notes (JL). With the permission of the participants all focus groups were digitally audio recorded and transcribed for the purpose of analysis.

Each focus group discussion lasted approximately 60 minutes and was attended by 5 to 6 participants. Using the pre-prepared discussion guide (Table 1) participants were invited to discuss their experience using the adjustable work station (e.g. general impressions of the sit-to-stand station; tasks undertaken while sitting and standing; cues to sit or stand and changes in work performance). At the completion of the focus group an incentive of a $10 AUD coffee voucher was given to each participant.

**Table 1.** Focus group discussion guide.

**1)**	**What do you think motivated you to be part of this study?**	
**2)**	**Thinking about the sit-to-stand workstation and how you used it —What were your general impressions of it?**	
	a)	Did it motivate you to change behaviour around sitting at work?
	b)	How much did you use the sit-to-stand workstation?
	c)	Was there anything about using the sit-to-stand workstation that you particularly liked?
	d)	Was there anything that you particularly disliked?
	e)	Any comments about the work surface attached to the workstation?
***Now I'd like to talk more specifically about the types of tasks you undertook in either the sitting or standing position.***		
**3)**	**Thinking back to your usual work day and the activities you usually do - What types of tasks did you generally do standing up?**	
Prompts		
	a)	Were there times of the day when you stood more?
**4)**	**Once again thinking back to your usual work day and the activities you usually do - What types of tasks did you generally do sitting down?**	
Prompts		
	a)	Were there times of the day when you sat down more?
**5)**	**Thinking about sitting and standing at work – what do you think made you change from sitting to standing?**	
	a)	Were there any reasons for those decisions?
Prompts		
	a)	You wanted to move more
	b)	Stiffness or pain
**6)**	**Were there any particular other things in the workplace surroundings that encouraged you to stand up more to complete your work?**	
Prompts		
	a)	Did you notice if other people in your work area around you were using a workstation?
	b)	Did certain types of footwear make it easier to stand up?
	c)	Did you find standing was enjoyable?
	d)	Were you able to stand for longer periods over time?
**7)**	**How long at a time did you tend to stand for?**	
**8)**	**Was there anything that stopped you from standing more than you did?**	
Prompts		
	a)	Did being in an open or closed office area make any difference?
	b)	Were there any tasks that were not practical while standing?
	c)	Was it comfortable to stand and work?
	d)	Did you have any injuries/pain/stiffness or other personal factors?
***Now let's move on to discuss whether you noticed any changes as a result of participating in the study in how you felt generally***		
**9)**	**Did anyone notice or experience any physical changes as a result of standing more?**	
Prompts		
	a)	Any changes in posture?
	b)	Any musculoskeletal changes?
	c)	Any changes in tiredness or energy levels?
	d)	Were these related to using the workstation?
**10)**	**What types of changes in your work performance did you notice?**	
Prompts		
	a)	Any effect on productivity?
	b)	Any effect on ability to concentrate?
**11)**	**Looking into the future and considering your experience with the sit-to-stand workstation -Would you continue to stand more often at work if you had access to an adjustable sit-to-stand workstation?**	
**12)**	**Would you like to have access to a sit-to-stand workstation?**	
Prompt		
	a)	Why/why not?
**13)**	**In closing, is there anything else you'd like to say about your experience of participating in the *Uprising Study* or about your experience of using a workstation or wearing the activity monitors?**	

### 2.4. Supervisor Interviews

Individual interviews (face-to-face (n = 9) and telephone (n = 3)) using a pre-prepared schedule (Table 2) were undertaken by one researcher (JL) and were of 30–40 minutes duration. Supervisors were invited to share their observations and perceptions in regard to their employee and the workstation (e.g. perceived advantages and disadvantages of the sit-to-stand station for their employee; personal perceptions of the sit-to-stand workstation; arguments for and against the sit-to-stand station in the workplace). Supervisor responses were recorded by hand and audio-recorded. At the completion of the interview an incentive of a $10 AUD coffee voucher was given to each supervisor.

**Table 2.** Individual interview discussion schedule.

***Thinking about Sit-to-Stand (STS) workstations and their place in the office environment (this is about how they fit into the workplace)***
Q1. What do you think are their advantages of having STS stations in the office environment?
(e.g. more room; more movement)
Q2. What do you think are their disadvantages?
( e.g. do they interfere with effective/productive workplace)
***Thinking about the staff who used the STS station:***
Q3. What advantages did the STS offer your staff member?
Q4. What disadvantages?
Q5. What did you (personally) like about them?
Q6. What did you (personally) dislike about the station?
Q7. Would you like all your staff to have STS station?
Q8. What would be your argument for getting STS's in your workplace?
Q9. What would be your argument against STS workstations?
***Thinking about sitting and standing at work, why did you agree for your staff member to be part of the study?***

## 3. Analysis

### Focus Groups and Individual Interviews

Focus group and individual interview notes and transcripts were analysed using an iterative process during and after the data collection to identify the main concepts. This study followed the items in the consolidated criteria for the reporting qualitative research (COREQ) checklist [35]. The focus group audio recordings were then fully transcribed verbatim by the research assistant (RT) and subjected to inductive open coding to identify emerging categories. The individual interviews were fully transcribed verbatim by the researcher (JL) and also subjected to inductive coding to identify emerging themes. The general inductive approach is a straightforward easily used, systematic set of procedures for analysing qualitative data and provides reliable findings [36]. The research assistant (RT) and one researcher (JL) performed the analyses independently, and then met to discuss and confirm key concepts for both sets of data. Employee and supervisor quotes to support themes were identified.

## 4. Results

A total of three focus groups (n = 17, 15 females and 2 males) and 12 (8 = females and 4 = males) individual interviews were completed. Table 3 presents the personal characteristics of the employees and supervisors.

**Table 3.** Personal characteristics of the employee and the supervisor.

Employee characteristics	Employees (n = 17)% (n)	
Age in years (mean ± SD)	34.8 ± 10.5	
Female (F)	94.0 (16)	
Body Mass Index (BMI)		
Under/healthy weight ^1^	82.0 (14)	
Overweight/obese ^2^	23.5 (4)	
Level of highest educational attainment		
Post graduate	88.0 (15)	
Other post school qualification	6.0 (1)	
Completed year 12 schooling or equivalent	6.0 (1)	
Smoking status		
Never smoked	88.0 (15)	
Ex-smoker	12.0 (2)	
Level on which workspace is located		
1^st^ or 2^nd^	82.4 (14)	
3^rd^ or 4^th^	17.6 (3)	

Supervisor characteristics	Supervisors (n = 12)% (n)	
Female (F)	67.0 (8)	
Employment status		
General staff (GS)	33.0 (4)	
Academic staff (AS)	67.0 (8)	
Level on which workspace is located		
1^st^ or 2^nd^	92.0 (11)	
3^rd^ or 4^th^	8.0 (1)	

^1^ BMI < 25; ^2^ BMI ≥ 25

### 4.1. Employee Findings

Qualitative analyses of the focus group data identified four concepts: (1) enhanced general well-being; (2) workability/practicality; (3) disadvantages of the retro-fit workstation; and (4) triggers to stand.

### 4.2. Enhanced General Well-being

Enhanced well-being was a strong theme highlighted by two-thirds of employees (n = 12). The employees indicated that they felt more energised at work, were less tired, more refreshed mentally and experienced a marked absence of back pain, as the following quotes illustrate:

*“I felt physically better and that helped mentally as well.”* (M, FGD1)

*“Alternating between sitting and standing really helped with lower back pain. Felt much better standing, less muscle tightness, more flexible. If I was already standing I'd be more likely to quickly do a stretch, it made initiating other movements easier.”* (F, FGD2)

*“I'm feeling so much more flexible…it's just really good to stretch, your body needs that stretch. I feel more energetic, less pains in my back, my neck, more flexible, more awake while working.”* (F, FGD3)

### 4.3. Workability and Practicality

When participants discussed workability/practicality they mentioned positive impacts that included less email exchanges with colleagues and more face-to-face interactions. Five participants indicated the flexibility to move around the office, and being able to stand resulted in more positive interactions with colleagues:

*“It was easier to move around, to go to talk to someone, to interact.”* (F, FGD1)

*“…had more people stopping in just to chat because I was standing, I think they came to talk to me more often, whereas in the past they may have sent an email.”* (F, FGD3)

Six participants commented on tasks and the practicality of standing versus sitting, as their decision to stand did not appear to disrupt their ability to concentrate on different work tasks:

*“I was surprised I could think on my feet and that I could do tasks that required concentration either sitting or standing.”* (F, FGD2)

*“I find I get a bit restless, usually I would daze off for a bit start fiddling around… now I could go and stand and I actually could actually keep doing what I was doing, you get that focus back.”* (F, FGD3)

However, there were feelings associated with being uncomfortable when standing in an open plan office. For example:

*“Movement could be distracting. Swaying when standing, thought if I did share my office with anyone it would be strange.”* (F, FGD2)

### 4.4. Disadvantages of the Sit-to-stand Workstation

When employees discussed the general impressions of the workstation, a common citing was the disadvantage of the retro-fitted workstation. Generally the comments related to the design of the workstation, and included: awkward to use; the monitor and keyboard position were uncomfortable; too much desk space taken up by the workstation; unstable keyboard platform when typing; and when standing chair placement was awkward in offices with restricted space.

*“... the temporary structure made the desk very unusable.”* (F, FGD1)

*“There was a disconnect between things on your desk and where you were ...”* (F, FGD1)

*“...hindered me doing the things I needed to do.”* (F, FGD3)

*“The keyboard moved up and down, I never felt comfortable.”* (F, FGD3)

*“Workstations are designed for standing not sitting so it was hard to find what to do with your chair.”* (F, FGD1)

### 4.5. Triggers to Stand

Employees mentioned a number of factors that prompted them to stand. These could be grouped into time-based prompts (e.g. downloadable apps that reminded you to stand), and task based factors (e.g. morning coffee, or after lunch when they felt less alert and had reduced capacity to concentrate as a cue to stand).

*“Used a timer, stood for ten minutes of every hour.”* (F, FGD2)

*“Morning tea with a coffee would stand, or start a new task.”* (F, FGD3)

*“I always want to nap at two o'clock so I stood up and that would really push me through.”* (F, FGD2)

Participants described more complex tasks, or those that required paperwork to be spread over the desk top as not conducive to standing. However, the majority of the participants agreed phone-calls and emails could be undertaken with ease whilst standing. Those who used a sit-to-stand workstation said leaving the workstation up at the end of the day acted as a standing trigger:

*“…left it up and the end of day, so would come in the next day and stand. Would stand for a large proportion of the morning as would come in fresh.”* (F, FGD2)

*“Others in my office with the same stand-up desk would encourage you to stand.”* (M, FGD1)

Of interest, six employees commented on the need for different footwear when standing. They changed shoes to go to meetings and other formal work functions, and/or wore either low heeled shoes, trainers or took shoes off when standing at their desk.

Finally, three of the participants requested that their workstation be removed. The reasons for removal included: too much movement in the keyboard and poor fit which reduced work efficiency; the nature of their work required access to the complete desk top and the workstation hindered this access; or they experienced acute neck pain.

### 4.6. Supervisor Findings

The supervisor interviews identified five concepts: (1) enhanced general health and well-being; (2) engagement with work; (3) flexibility to move more in the workplace; (4) employee morale; and (5) injury prevention and management.

#### 4.6.1. Enhanced General Health and Well-being

A recurring theme amongst supervisors was the potential of the workstations to increase general health and well-being of their employees, with seven supervisors emphasising back health care. They suggested that the workstations opened up a dialogue about posture and occupational considerations for good health. An illustration of these follows:

“…it started a conversation about health/footwear/back care/preventing back pain.” (GS, F)

*“Yes much better for back health.”* (GS, F)

*“General health benefits, encourages people to talk about health/posture and exercise.”* (GS, F)

*“…very positive for the staff member, [she] is a Team Leader and seemed very productive… Staff member has a back issue and helped with that.”* (GS, M)

*“Health benefits, occupational health, posture, back issues, the evidence supports it to improve employee overall health.”* (AS, F)

#### 4.6.2. Engagement with Work

Supervisors described observing an increased level of engagement by employees with their work after the installation of the sit-to-stand work desks. For example:

*“…very positive for the staff member, [she] is a Team Leader and seemed very productive.”* (GS, M)

*“…he seemed like he was enjoying work more, he was more focused and happier.”* (GS, F)

*“…employee outputs plus, plus, positivity, mental difference in staff member, they appeared more alert, sharper, outputs increased.”* (GS, F)

*“…concentration is greater, less sluggish, the mind is active.”* (AS, F)

Two supervisors described the sit-to-stand work stations as having a negative impact on the employee and their workability, as the following quotes illustrate:

*“…was easily distracted, productivity decreased and mostly sat at desk.”* (GS, M)

*“Less inviting if a receptionist is standing for the customers.”* (GS, M)

#### 4.6.3. Opportunity and Flexibility for Staff to ‘Move More’

Supervisors cited this was an opportunity to promote more movement in the office space, and that the workstations worked in tandem with other public health messages aimed at promoting physical activity in the work place, such as ‘take the stairs, not the lift’. In addition some supervisors already had strategies in place to break up prolonged periods of sitting, such as ‘walking emails’, hence the sit-to-stand workstations complemented these.

*“We use walking emails so this reinforced the message of move more, we want to get people in our organisation to stand more so this was key.”* (AS, F)

*“…did not interfere with space in office. No problems for staff or the office at all, more positive work space.”* (GS, F)

*“Past health issues may have improved, opportunity to move more and incidental physical activity was a positive.”* (GS, F)

#### 4.6.4. Valuing Employees and Morale

Supervisors discussed the notion of staff as a ‘valuable resource’, and therefore the need to provide a supportive working environment. This included strategies to ensure staff felt valued and respected and supporting the creation of an organisational policy that enables all staff to access a standing station. For example:

*“If you value and respect your staff they will be productive. They spend more time at work than home so [we] need to be investing in employee health and well-being and reward what they do in the workplace by having the option to stand.”* (AS, M)

*“It is beyond just health, it is looking after your staff, caring for them and making sure they are feeling valued.”* (AS, F)

A few supervisors described the change in mood when the sit-to-stand desk was removed:

*“There was a sense of mourning when the desks were removed. A real lull.”* (GS, F)

*“Was a sense of loss when removed - staff member said she missed it!”* (AS, M)

#### 4.6.5. Injury Prevention

Many supervisors reported that injury prevention and the implications for occupational health were important considerations for their organisation. They also suggested a comprehensive educational package and/or instructional video (pre- and post the workstation) be made available, and an ergonomic assessment pre-and during the work station installation. Other comments highlighted office-space design and cost-benefit analysis prior to the installation of the work station as important considerations for an organisation.

*“There are no negatives for getting sit-to-stand workstations in the workplace but design, fit and an ergonomic assessment and occupational health are all key issues.”* (AS, F)

*“The benefits balance out any costs, and I can only see benefits, this is a best buy for Curtin.”* (GS, M)

*“…duty of care issues, health benefits, injury management and flexibility for staff.”* (GS, M)

However, whilst it may be cost prohibitive for some workplaces, one interviewee stated:

*“Cost is not an issue, this is the next workplace challenge and we need to tackle it.”* (AS, F)

## 5. Discussion

The Uprising study explored the perceptions and experiences of office based employees together with the supervisor's perception of the adjustable workstation in the workplace. This is a novel approach, as to our knowledge no other research has been conducted into both the employee and their immediate supervisor's perception of the adjustable workstations. It provides valuable information for organisations and occupational health promotion practitioners interested in integrating environmental strategies into the workplace to break up prolonged periods of sitting.

### 5.1. Employee Perceptions

The employees' responses highlight the general health benefits from this relatively short four week intervention. The perceived advantages included: increased physical and mental health; increased flexibility; and opportunity to stretch which, in turn, increased the opportunity to move more, and sit less; and interact with others in the workplace. It was expressed that the sit-to-stand station created a real ‘energy’ in work spaces. Overall, the employees seemed to embrace the sit-to-stand workstation, which is consistent with the recent literature that indicates they are well received, have high usability and acceptability [16],[19],[23],[25], with few employees requesting the removal of the sit-stand station once installed. Other Australian research has reported employees were committed and frequent users of the standing option once given the opportunity to do so [19]. Furthermore, the Uprising study found the commitment to standing was influenced by the perception of improved productivity and/or experience of a health benefit, as previously reported in the literature [18],[19],[23],[37],[38].

### 5.2. Supervisor Perceptions

General health and well-being, and the need for a positive impact on workers dominated the supervisor perspective in this study. The supervisors emphasised injury prevention and employee morale as two important by-products of the adjustable workstation. These two considerations were not mentioned by the employees, but were seen as an integral component of duty of care by supervisors. Recently, reducing sedentariness [4], together with musculoskeletal [39] and mental health [40] have grown as occupational health issues. Supervisors' consistently acknowledged that their workers spent almost one third of their adult life at work, and accordingly they wanted their employees to feel cared for and valued. Pronk [2] also reported that a higher level of satisfaction with the workplace environment, the more likely employees will be engaged, productive and have less sick-days, and move more after installation of sit-to-stand workstations [2]. Future workplace design that aims to intentionally reduce sitting time has the potential to impact on employee health and productivity.

### 5.3. Workplace Champions, Role Models and the Business Case

The supervisors' suggested practices to support increased movement such as a ‘work place champion’ or ‘role model’ could be used to promote the standing stations, together with other opportunities to be break up sitting time in the workplace, such as walking emails and standing meetings. The supervisors' believed the champions or role models could act as change agents, advocating for the allocation of resources and influence organisational policy to target sedentary behaviour in the work place. Of interest, it has been demonstrated that supervisors are important catalysts for cultural change in the work place [41]. Furthermore, the supervisors recognised that despite an initial cost outlay, the installation of sit-stand workstations offer an opportunity for organisations to promote an ethos that is flexible, open to change, and supportive of their staff [42]. Contemporary health promotion practice stresses the importance of structural environmental change to encourage behaviour change [43]–[45].

Finally, supervisors highlighted the need for a business case for the purchase, installation and supporting strategies (e.g. periodic ergonomic assessments). This would ensure equitable access to the adjustable workstation by all employees and send a message that the health of employees is valued. Gilson et al. 2012 [38] cited the costs and employer financial constraints as a possible limiter when allocating adjustable workstations to all employees. In addition there has been a call for larger scale and longer term studies in the work place to establish the cost effectiveness of sit-stand desks and provide a basis for the business case for implementing them for all [37].

### 5.4. Opportunities, Loss and Triggers to Stand

The findings from both the employees and the supervisors reinforce the notion of choice and opportunity to move more and be flexible in the work place were a welcome option beyond just the health and potential musculoskeletal benefits. Employees indicated that there were a range of simple tasks that could be completed standing (e.g. checking emails and making phone calls) while other tasks which involved greater concentration, such as reading, marking student assessments, and writing, were better suited to sitting. Depending on the stability of the keyboard, data entry and tasks that require many key strokes were not suited to the workstation used in this study, as they had too much keyboard instability. Overall, some tasks, such as large volume data entry and certain roles (e.g. customer service that requires eye contact with clients), may be less suitable for a standing desk. Interestingly, both the supervisors and employees mentioned a ‘mourning period’ when the sit-to-stand stations were removed, suggesting the value attached to the workstations by the employee.

### 5.5. Disadvantages

There were a number of disadvantages of the retro-fit sit-stand station identified in the study, and these are consistent with the literature [19],[38]. These included the downward movement of the Ergotron workstation hinged arm, and the keyboard platform provided challenges for some participants in regard to bounce and lack of stability. The distance and tilt of the screen were also problematic for some employees, along with the limited table space on the station. These disadvantages could be easily remedied through the installation of full desk tops that move up and down, which are more stable and functional.

### 5.6. Strengths and Limitations

The Uprising study explored both employees' and their supervisors' perceptions, whilst other Australian and international studies have been limited to the employees' experience only. Strengths of the study are real-world applicability as we used a natural setting in a large organisation [19] and the whole of office approach whereby the desks were the employees' own desks. However, the study only recruited small numbers and some individual interviews were completed by telephone due to their external work commitments. One other key issue may have included the open workspace layout and the intervention participants' knowledge of the impact of prolonged sitting in the workplace.

## 6. Conclusion

The results of this study suggest sit-to-stand workstations appear to be effective in breaking up prolonged sitting time, improving work performance, improving mood, and positively influencing some health outcomes. To date the literature has few examples of qualitative research, and to our knowledge no studies with both the supervisors' and employees' views and observations. Sit-to-stand desks represent just one strategy for reducing prolonged occupational sitting time however, they need to be part of a comprehensive approach. These findings hold public health significance, as reducing prolonged sitting time is an important contributor to the long term goal of a more active adult population.
